# Proceedings of the Third Annual Conference of the MidSouth Computational Biology and Bioinformatics Society

**DOI:** 10.1186/1471-2105-7-S2-S1

**Published:** 2006-09-26

**Authors:** Jonathan D Wren, Yuriy Gusev, Andrey Ptitsyn, Stephen Winters-Hilt

**Affiliations:** 1 Advanced Center for Genome Technology, Stephenson Research and Technology Center, Department of Botany and Microbiology, 101 David L. Boren Blvd., The University of Oklahoma, Norman Oklahoma 73019, USA; 2 Department of Surgery, Health Sciences Center, The University of Oklahoma, Oklahoma City, Oklahoma 73104, USA; 3 Department of Microbiology, Immunology and Pathology, Colorado State University, Fort Collins, CO 80523-1619, USA; 4 Department of Computer Science, University of New Orleans, New Orleans, LA, 70148, USA and The Research Institute for Children, 200 Henry Clay Ave., New Orleans, LA 70118, USA

## Introduction

The Third Annual MidSouth Computational Biology and Bioinformatics Society (MCBIOS-III) conference was held in Baton Rouge, Louisiana on March 2^nd^-4^th^, 2006, under the banner of "Bioinformatics: A Calculated Discovery". The conference featured three days of scientific platform presentations, posters, and panel discussions, with a Cheminformatics satellite conference on the final day. The conference resulted in the 22 papers shown in this proceedings, almost doubling the number over last year's 12 papers [1-12], which is consistent with the rapid growth of MCBIOS since its inception [13]. The strong growth in the Society's proceeding publications, and the strong showing of over 100 presenters at MCBIOS-III, is all the more impressive given the last-minute change of venue forced by the impact of Hurricane Katrina on the Gulf Coast region.

At MCBIOS 2006, awards for outstanding oral presentations were given to the following students: Yuanyuan Ding of the University of Mississippi (1^st ^place), Stephanie Hebert of the University of Arkansas at Fayetteville (2^nd ^place), and Michael Dyar of Ouachita Baptist University (3^rd ^place). Awards for outstanding poster presentations were given to the following students: Zhijie Jiang of Louisiana State University (1^st ^place), Charles McChesney of the University of New Orleans (2^nd ^place), Vinay Ravindrakumar of UALR/UAMS (3^rd ^place).

## Proceedings summary

Papers submitted to these proceedings were peer-reviewed by at least two reviewers, including program committee members and external experts as necessary. The aim of the proceedings was to be inclusive yet rigorous in selecting only high-quality papers, with the final acceptance rate being 73%. The innovative bioinformatics research in the region is reflected in these accepted papers, which fall into several general themes as follows:

## Advances in methods for microarray analysis

There are a number of steps in microarray analysis and the papers published here are indicative of the impact of computational methods at each one. The first step is technical – getting good empirical measurements. Tao Han *et al. *present a technical study on the best methods to optimize washing and hybridization conditions for microarrays[14], and showed that optimizing these conditions improved accuracy and reproducibility.

After microarray data has been gathered, the next challenge is to determine the statistical significance of the transcriptional response. Robert Delongchamp *et al. *present a new method for computing the overall statistical significance of a treatment effect among predefined sets of genes (e.g., sets of genes grouped by gene ontology (GO) terms) [15]. Computer simulations demonstrated that ignoring the correlations among genes overstates the significance assigned to GO terms. The authors propose statistical tests based upon meta-analysis methods for combining p-values to correct for gene expression correlations.

After defining which genes are differentially expressed, the next step is often grouping or clustering these genes into similar expression profiles. Raja Loganantharaj *et al. *measured the effectiveness of microarray clustering algorithms [16] by calculating inter-cluster cohesiveness and intra-cluster separation for biological processes and molecular functions associated with the genes in the cluster. In a similar effort, Ding and Wilkins introduce a variant of the Recursive Feature Elimination (RFE) method for classifying gene expression data [17]. Their method is implemented using a Support Vector Machine (SVM), and borrows ideas from simulated annealing to reduce what is normally a very computationally intensive task. RFE is a common and well-studied method for reducing the number of attributes used for further analysis or development of prediction models. The goal of the algorithm is to improve the computational performance of recursive feature elimination by eliminating chunks of features at a time with as little effect on the quality of the reduced feature set as possible. The algorithm was tested on several large gene expression data sets and shown to be more time efficient in generating a set of attributes that is very similar to the set produced by RFE.

Data produced in microarray experiments carries a high degree of stochastic variation, and in time series data, this variation can obscure periodic patterns. Furthermore, in many experiments a limited number of replicates covers no more than two complete periods of oscillation. To address this, Andrey Ptitsyn *et al. *developed a new method for identifying periodicity within time-series data and compared its performance versus previous methods of identifying periodicity to show that their new method was more sensitive and precise [18]. The authors applied this method to a study of circadian expression on a large data set, representing three different peripheral murine tissues, and re-analyzed a number of similar time series data sets produced and published independently by other research groups over the past few years. This test is based on a random permutation of time points in order to estimate the non-randomness of a periodogram. This Permutated time, or Pt-test, is able to detect oscillations within a given period in expression profiles dominated by a high degree of stochastic fluctuations or oscillations of different irrelevant frequencies. The software is implemented as a set of C++ programs available from the authors on the open source basis.

Finally, post-response microarray analysis typically consists of identifying functional commonalities among the responding genes. Towards this end, Hongmei Sun *et al*. report a new FDA microarray analysis tool called Gene Ontology For Functional Analysis (GOFFA) [19], which provides an interface for visualization and analysis of GO categories associated with responding genes.

## Microarray studies

Bioinformatics entails not just the development of new methods of microarray analysis, but studies on the effectiveness of their application. To this end, several groups report bioinformatics-based microarray analysis using several model systems. Nan Mei *et al*. report their analysis of liver-based gene expression changes of Big Blue transgenic rats when fed comfrey, a perennial plant native to most of North America, Europe, and western Siberia that has been used as a herbal medicine for more than 2000 years [20]. Their study of gene expression profiles helps provide a better understanding of hepatotoxicity induced by comfrey and exerted through pyrrolizidine alkaloid plant components.

Lei Guo *et al. *used microarray analysis to study primary hepatocytes from mice that had been exposed to peroxisome proliferators-activated receptor alpha (PPAR-alpha) agonists [21]. PPAR-alpha agonists lower plasma triglyceride and cholesterol levels, which is a very important pharmacological effect given the rise in cholesterol-related heart deaths as well as obesity in western societies. Their results suggest that PPARα agonist exposure results in increased oxidative stress and increased peroxisome proliferation, which can account for the pleiotropic and sometimes carcinogenic effects of PPAR-alpha agonists.

Tao Han *et al. *report a study of L5178Y mouse lymphoma cells, using large and small colony Thymidine kinase mutants[22]. To gain insight into the underlying mechanisms for formation of large and small colony *thymidine kinase *(*Tk*) mutants due to different growth rates of the mouse lymphoma cells, the authors conducted microarray analysis of gene expression profiles from the two different types of mutants. Their findings suggest that genes in the DNA segment altered by the *Tk *mutations were significantly up-regulated in the small colony mutants, but not in the large colony mutants, leading to differential expression of a set of growth regulation genes related to cell apoptosis and other cellular functions related to the restriction of cell growth.

Tao Chen *et al. *contrasted the effect of aristolochic acid (AA) upon different organ systems – liver versus kidney[23]. AA is an active component of herbal drugs and can induce nephropathy and kidney cancer in people and rodents, but does not damage the liver. To evaluate whether microarray analysis can be used for distinguishing the tissue-specific carcinogenicity of AA, they examined gene expression profiles in kidney and liver of rats treated with carcinogenic doses of AA. They found many more significant genes associated with carcinogenesis, defense response, apoptosis, immune response and organic acid metabolism in kidney than in liver due to AA exposure. These differential alterations between kidney and liver could be the underlying mechanisms for the tissue-specific toxicity and carcinogenicity of AA.

## Machine learning-based cheminformatics

Stephen Winters-Hilt's group reports several studies in cheminformatics. In Winters-Hilt [24] the author describes Hidden Markov Model (HMM) variants based on hash and/or gap interpolated Markov models, and a novel implementation of HMM-with-Duration is introduced. The new HMM-with-Duration implementation is much simpler than those previously known and has far-reaching application to gene-structure identification and analysis of channel current blockade data. In Winters-Hilt *et al. *[25] they describe SVM implementations for clustering and classification, where novel, information-theoretic, kernels were successfully employed for notably better performance over standard kernels, and where two SVM approaches to multiclass discrimination/classification are described: (i) internal multiclass (with a single optimization), and (ii) external multiclass (using an optimized decision tree). In Iqbal *et al. *[26] they apply Adaboost to circumvent the typical limitations in decision tree approaches, at the expense of requiring an expert to train the classifier (i.e., there is minimal automation in the tuning of key parameters). The approach is based on feature primitives and, once tuned on a given data-type, results in the dramatic reduction in training time on a given data-set to find the best solution.

In Winters-Hilt [27] a nanopore cheminformatics method is described that is able to measure molecular conformational and binding characteristics by use of a reporter molecule that binds to certain molecules, with subsequent distinctive blockade by the bound-molecule complex. A web-interface to many of the machine learning based cheminformatics methods that have been developed is also described. It is hypothesized that reaction histories of *individual *molecules can be observed on model DNA/DNA, DNA/Protein, and Protein/Protein systems, and preliminary results are shown to support this for each case. Nanopore detection capabilities are also described for highly discriminatory biosensing, binding strength characterization, and rapid immunological screening. The author suggests that the heart of chemistry is now accessible to a nanopore-based, single-molecule, observation method that can track both external molecular binding states, and internal conformational states. In Winters-Hilt *et al. *[26] the nanopore detector biophysical advances from [27] and machine learning pattern recognition methods from [24,25] are used to help systematically explore internal DNA dinucleotide flexibility, with particular focus on HIV's highly conserved (and highly flexible/reactive) viral DNA termini. To support this effort a new, HMM-based, filtering method is introduced that amplifies emission variances in the HMM to achieve level-projected filtering for ease of kinetic feature extraction from the observed channel blockade currents. The observed state kinetics of the DNA hairpins containing the CA/TG dinucleotide provides strong evidence for HIV's selection of a peculiarly flexible/interactive DNA terminus.

Jonathan Wren implemented and tested a machine-learning method for automated recognition and extraction of chemical names within text [28]. The method was tested on over 7 million abstracts, which is unusually large as far as most text-based testing datasets go, yet was important to demonstrate the scalability of the approach and show that it might be feasible as a method to automatically identify chemicals within text. The study was also able to pair chemical name variants together and study how these spelling variants affected information retrieval in PubMed and Ovid, demonstrating that document recall for chemical names is sensitive to the exact spelling of the term used.

## Databases

Databases are an integral part of modern biomedical research, helping to both locate and analyze categorical data. Thodima *et al. *[29] describe the RiboAptDB, a comprehensive source for sequence information on ribozymes and aptamers. Such 'unnatural' *in vitro *data are not represented in the public 'natural' sequence databases such as GenBank and EMBL. As with the sequence information found in nature, however, the amount of sequence data generated by *in vitro *selection experiments has been accumulating exponentially. In their latest version of RiboaptDB there are 370 artificial ribozyme sequences and 3,842 aptamer sequences. The authors' database also includes numerous functions, such as a general search feature, an individual feature-wise search, and a user submission form for new data through online and also local BLAST search.

Nahum *et al. *presented EGenBio, a web-based data management system for studies in Evolutionary Genomics and Biodiversity [30]. It includes managed access to curated data from external databases, rapid manipulation of sequences, alignments, and trees, and integration and visualization of outputs. EGenBio was developed around their research program on comparative genomics and a pilot mithochondrial genome database. It is freely available at .

## Genomic Analysis

Evolution often proceeds through gene duplication and subsequent functional divergence, and identifying the relationships between gene families is an important part of studying and understanding the difference between species. To aid in this, Ron Frank *et al. *report an automated method of identifying gene family members in plants through a battery of software tools [31], making the process simpler and more rapid.

Alexander Kel *et al. *tackle an important problem in identifying common transcription factor binding sites from microarray data [32]. The authors developed a novel computational approach for revealing key transcription factors by knowledge-based analysis of gene expression data with the help of databases on gene regulatory networks. They demonstrate that promoters of genes encoding components of many known signal transduction pathways are enriched with binding sites of those transcription factors that are endpoints of the considered pathways. Application of the approach to microarray gene expression data on TNF-alpha stimulated primary human endothelial cells helped to reveal key transcription factors that may explain concerted expression changes in signal transduction networks. The corresponding software and databases (TRANSFAC^® ^and TRANSPATH^®^) are available at .

## Miscellaneous

Smolinski *et al. *[33] report an independent component analysis-motivated approach to classify cortical evoked potentials. They analyze data deriving from experiments based on measuring neural activity in a controlled setting (i.e., normal) as well as under exposure to some external perturbation (nicotine exposure). They describe a practical implementation of the method, based on hybridization of independent component analysis, multi-objective evolutionary algorithms, and rough sets.

Nagarajan *et al*. [34] report a novel method to identify antimicrobial activity among peptide motifs. They use a Fourier transformation based method to mine the peptide space for potential antimicrobial activity. They also demonstrate that a property-based coding approach can significantly boost the characteristic properties of biomolecules.

## Future Meetings

The fourth annual MCBIOS Conference will be held in New Orleans, Louisiana, February 1–3, at the University of New Orleans' Lindy Boggs International Conference Center. Our web site, , contains further information on the society and future meetings. MCBIOS is a regional affiliate of the International Society for Computational Biology .

## Authors' contributions

All authors served as co-editors for these proceedings, with JDW serving as Senior Editor. All authors helped write this editorial.
